# Multi-trajectories of systolic and diastolic hypertension and coronary heart disease in middle-aged and older adults

**DOI:** 10.3389/fpubh.2022.1017727

**Published:** 2022-11-24

**Authors:** Mingzhuo Li, Miao Zhou, Yang Yang, Yafei Liu, Chaonan Yin, Wenting Geng, Chunxia Wang, Fang Tang, Yang Zhao, Fuzhong Xue, Xiubin Sun, Zhongshang Yuan

**Affiliations:** ^1^Center for Big Data Research in Health and Medicine, The First Affiliated Hospital of Shandong First Medical University & Shandong Provincial Qianfoshan Hospital, Jinan, China; ^2^Office of Hospital Level Review, Shenzhen Longhua District Central Hospital, Shenzhen, China; ^3^Health Management Center, Affiliated Hospital of Jining Medical University, Jining, China; ^4^Department of Biostatistics, School of Public Health, Nanjing Medical University, Nanjing, China; ^5^Department of Biostatistics, School of Public Health, Cheeloo College of Medicine, Shandong University, Jinan, China; ^6^Institute for Medical Dataology, Cheeloo College of Medicine, Shandong University, Jinan, China

**Keywords:** multi-trajectory, systolic hypertension, diastolic hypertension, coronary heart disease, blood pressure management

## Abstract

**Objective:**

This study aimed to investigate multi-trajectories of systolic and diastolic hypertension and assess their association with the risk of coronary heart disease (CHD) in middle-aged and older Chinese adults.

**Methods:**

The study cohort comprised 4,102 individuals aged 40–75 years with records of at least four systolic blood pressure (SBP) and diastolic blood pressure (DBP). A group-based multi-trajectory model was adopted to identify multi-trajectories of systolic and diastolic hypertension, followed by a logistic model to assess the independent associations between these trajectories and CHD risk. The multinomial logistic model was used to evaluate the impact of baseline covariates on trajectory groups.

**Results:**

Six distinct trajectories for systolic and diastolic hypertension were identified which represent distinct stages of hypertension and were characterized as low-stable, low-increasing, medium-decreasing, medium-increasing-decreasing, isolated systolic hypertension phase, and high-decreasing. Compared with the low-stable group, the adjusted odds ratios (ORs) and 95% confidence intervals (CIs) were 2.23 (1.34–3.70) for the medium-increasing-decreasing group and 1.87 (1.12–3.11) for the high-decreasing group after adjustment for baseline covariates. Compared with the low-increasing group, the ORs and 95% CIs were 1.88 (1.06–3.31) for the medium-increasing-decreasing group. Age, gender, drinking, body mass index (BMI), triglyceride (TG), and fasting plasma glucose (FPG) were independent predictors for trajectory groups 4 and 6.

**Conclusion:**

Novel, clinically defined multi-trajectories of systolic and diastolic hypertension were identified. Middle-aged and older adults with medium-increasing-decreasing or high-decreasing blood pressure trajectories are potentially critical periods for the development of CHD. Preventing adverse changes in hypertension status and reducing the high risk of CHD is necessary for people in distinct trajectory groups.

## Introduction

High blood pressure (BP) is an important, well-established, modifiable risk factor for coronary heart disease (CHD) (1, 2). From 2012 to 2015, the prevalence of hypertension was 44.60, 55.70, and 60.2% in Chinese adults aged 45–54, 55–64, and ≥75 years, respectively (3). Moreover, the awareness of hypertension in China was <50% (3, 4). In one study, fewer than a third of Chinese adults aged 35–75 years with hypertension were being treated and BP levels were controlled in fewer than one in twelve (4). High BP needs to be successfully managed to prevent subsequent CHD.

There are limitations to only considering BP at a single time point. For example, the risk of CHD likely differs between two individuals with the same BP at baseline, if the BP increases in one but decreases in the other during follow-up. Studies of BP on only one occasion (5, 6) cannot determine the effect of variations in BP on the risk of cardiovascular disease. Fortunately, some recent studies investigated trajectories of BP, including trajectories of systolic blood pressure (SBP) alone, diastolic blood pressure (DBP) alone, and mid-BP (calculated as [SBP+DBP)/2) (7–12). However, several issues have not yet been adequately addressed. First, patterns of co-evolution in both SBP and DBP have been rarely investigated. Because there are often correlations between SBP and DBP, a single indicator trajectory of SBP or DBP is insufficient to capture the potential synergistic effect of SBP and DBP on CHD. Second, longitudinal changes in hypertension status have not been well-studied despite medical guidelines emphasizing thresholds for adequate control of BP (13–15). Compared with continuous BP, patterns of changes in hypertension status may have novel significance for predicting CHD risk. Third, recent research studies have rarely resulted in recommendation for the prevention of CHD in people with different trajectories (7, 10–12, 16, 17). From a prevention perspective, individuals in BP trajectories with high CHD risk need to change their adverse trajectories in time.

The cohort of the present study comprised healthy Chinese adults aged 40–75 years at baseline for whom records of repeated measurements of SBP and DBP during follow-up were available. Using a multivariate group-based trajectory model (GBTM), we determined multi-trajectories of systolic and diastolic hypertension and then used logistic regression analysis to assess associations between the identified trajectories and risk of CHD, followed by adjusting for different baseline covariates. We assumed that hypertension trajectories can be changed and formulated prevention recommendations for each of the identified types of trajectory to prevent individuals from transitioning to trajectories with higher risks of CHD.

## Materials and methods

### Study cohort and design

The cohort for this study was recruited from individuals having routine health checks at the Health Management Center of the Affiliated Hospital of Jining Medical University. The study protocol was approved by the ethics committee of the School of Public Health, Shandong University (No. 20031112). Written informed consent was obtained from all participants.

The initial group recruited comprised 14,895 participants aged between 40 and 75 years at baseline with health check records from 2007 to 2015, multiple measurements during follow-up, and no evidence of CHD at baseline. Body mass index (BMI), SBP, DBP, low-density lipoprotein cholesterol (LDL-C), high-density lipoprotein cholesterol (HDL-C), triglyceride (TG), and fasting plasma glucose (FPG) were averaged for analysis if individuals had more than one follow-up visit within a single year. The first health check year for each individual was taken as the baseline by left aligning the subsequent follow-up years. For example, the follow-up years of an individual who had health checks in 2008, 2009, and 2014 were specified as 1, 2, and 7, respectively. There were 13,447 individuals with complete baseline data for BMI, SBP, DBP, LDL-C, HDL-C, TG, FPG, smoking, and drinking; of whom, 4,102 had at least four recorded SBP and DBP measurements. All information on SBP and DBP was obtained before the diagnosis of CHD or the end of follow-up. We investigated multi-trajectories of systolic and diastolic hypertension together with their association with the risk of CHD to identify longitudinal trends in hypertension and enable guidance regarding the early prevention of CHD.

### Examinations

All participants underwent basic physical examinations including height, weight, and BP. Height and weight were measured with the participant wearing light clothing and barefoot. BMI was defined as the weight (kg) divided by the square of the body height (m). Trained medical staff measured the BP three times in the right upper arm with the participant seated and after a 5-min rest. Smoking was defined as a history of or current smoking of cigarettes. Drinking was defined as a history of persistent alcohol intake or current excessive drinking. Peripheral blood samples to measure LDL-C, HDL-C, TG, and FPG were obtained after overnight fasting for 12 h (18). Similarly to previous studies (19–21), we used averaged levels of SBP and DBP for individuals with more than one recorded BP in a single year. Systolic hypertension was defined as SBP ≥140 mmHg, and diastolic hypertension was defined as DBP ≥90 mmHg (13, 19).

### Definition of CHD

The health check-up data were linked with records from the Medical Insurance Office, hospital admissions, and the Provincial Center for Disease Control in Shandong Province. Patients with CHD were identified in accordance with the International Classification of Diseases, 10th revision International Classification of Diseases (ICD-10) clinical codes, including I20, I21, I22, I23, I24, and I25 (22), which denote angina pectoris, acute myocardial infarction, subsequent myocardial infarction, complications after myocardial infarction, other acute ischemic heart diseases, and chronic ischemic heart diseases, respectively. The earliest date of diagnosis of CHD in any of these records was considered the date of the outcome of CHD (18). Medical insurance records were available up until 14 September 2016.

### Statistical analyses

For continuous variables, normal data were presented as means ± standard deviations (SDs), whereas non-normal data were presented as median (interquartile ranges) and categorical variables as numbers (proportions). ANOVA or the Kruskal–Wallis test was used to compare continuous variables and the chi-square test to compare categorical variables. Latent mixture modeling (PROC TRAJ) was used to create a group-based multi-trajectory model to determine trajectory patterns of systolic and diastolic hypertension jointly (23–25). The multivariate trajectory model could conceptually capture the correlation and the potential synergistic effect of systolic and diastolic hypertension. Multi-trajectories of systolic and diastolic hypertension mean that each one in a group has two trajectory curves, one being for systolic and the other for diastolic hypertension. To ensure the accuracy and stability of the trajectory curves, we included only individuals with at least four recorded SBP and DBP measurements. One to seven trajectories were fitted using the logistic regression with a linear and quadratic trajectory function, which also outputted the posterior probability of each individual belonging to each specific trajectory group. We selected models with the smallest absolute value for Bayesian information criteria (BIC), the average posterior probability of each trajectory group being required to be ≥70%.

A logistic regression model was used to evaluate the association between multi-trajectory groups and the risk of CHD with adjustment for gender, baseline age, smoking, drinking, BMI, LDL-C, HDL-C, TG, and FPG. Because the high censoring rate (95.64%) may have led to a biased estimate in a Cox model (26), we used a logistic regression model, as described in the previous study (7). We specified different trajectories as references to identify all groups that differed significantly. Finally, groups 1 and 2 were, respectively, considered reference groups.

The multinomial logistic model was used to evaluate the impact of baseline covariates on trajectory groups. The effect of single covariates and multiple covariates on trajectories with trajectory group 1 as a reference was evaluated. Because women had a low rate of smoking, we constructed one multinomial logistic model without gender and another model without smoking when adjusting multiple covariates.

All analyses were performed with SAS 9.4 and R 4.0.2. Two-sided *P*-values <0.05 were statistically significant.

### Sensitivity analyses

Sensitivity analyses were performed to make the findings more convincing. To highlight the advantage of the multivariate trajectory model, we used systolic hypertension status and follow-up time points to fit univariate trajectories of systolic hypertension, used the same process to fit univariate trajectories of diastolic hypertension, and further assessed the relationship between univariate trajectories and CHD.

## Results

During the follow-up period, 179 cases of CHD were identified in 4,102 individuals aged 40–75 years at baseline. The CHD incidence density (per 1,000 person-years) was 6.24, with the median number of records for systolic and diastolic hypertension of 6 (ranging from 4 to 9) and the median follow-up time of 7.09 years (ranging from 2.79 to 9.65).

**Figure 1:**
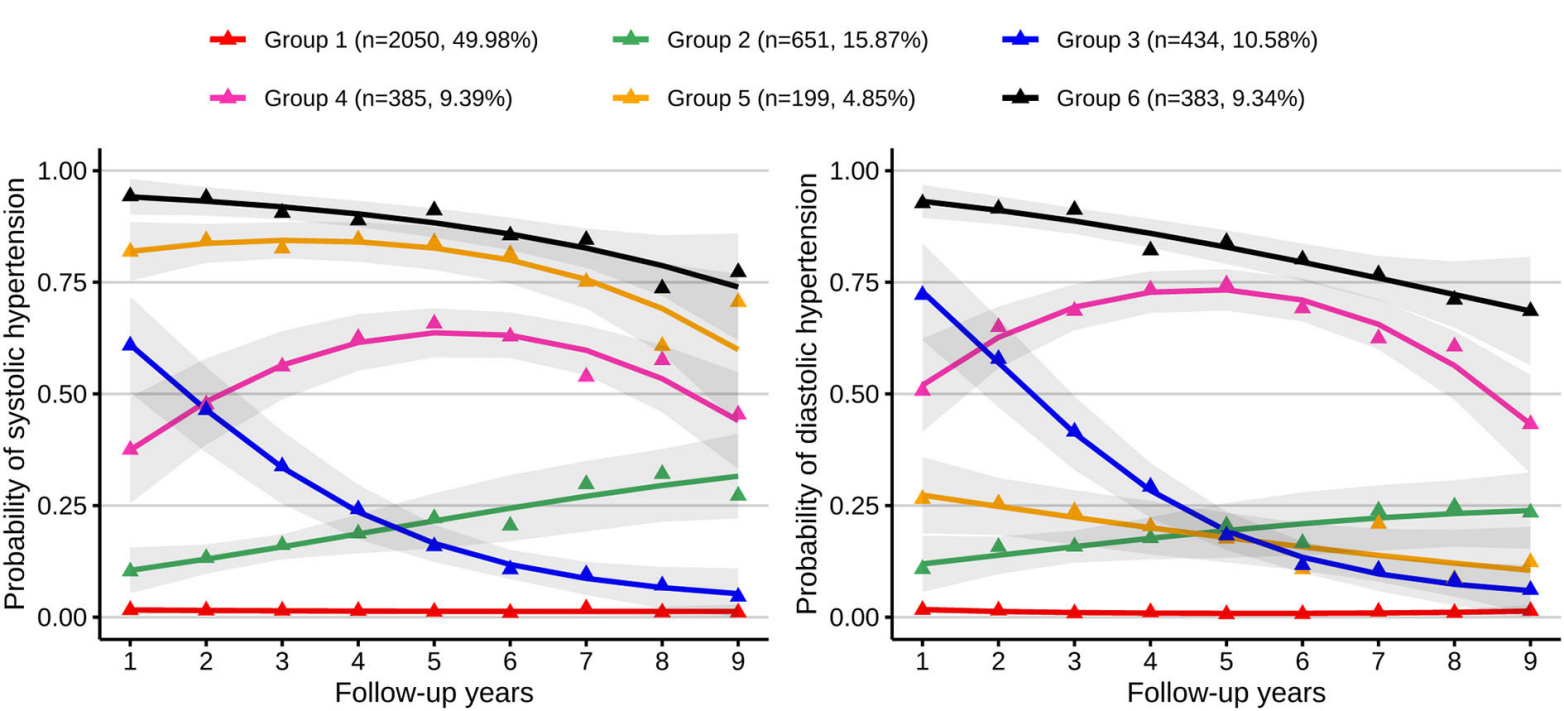
Multi-trajectories of systolic and diastolic hypertension. Figure shows six multi-trajectories of systolic and diastolic hypertension which were identified from GBTM. Gray shadows represent the 95% confidence intervals for each trajectory; the solid lines represent expected trajectories; the points represent observed trajectories; numbers and proportions of group memberships are listed. Group 1 indicates the low-stable group; group 2, the low-increasing group; group 3, the medium-decreasing group; group 4, the medium-increasing-decreasing group; group 5, the isolated systolic hypertension phase group; group 6, the high-decreasing group.

Supplementary Table S1 presents the results of fitting a GBTM. A model with six trajectory groups had optimal BIC with average posterior probabilities ≥70%. Details of variables for these six trajectories are listed in Supplementary Table S2. Figure 1 shows the six multi-trajectory curves of systolic and diastolic hypertension. Trajectories were labeled as group 1 (low-stable, *n* = 2,050, 49.98%), group 2 (low-increasing, *n* = 651, 15.87%), group 3 (medium-decreasing, *n* = 434, 10.58%), group 4 (medium-increasing-decreasing, *n* = 385, 9.39%), group 5 (isolated systolic hypertension (ISH) phase, *n* = 199, 4.85%), and group 6 (high-decreasing, *n* = 383, 9.34%). The average posterior probabilities for these six trajectory groups were 89.47, 72.12, 75.51, 75.61, 78.52, and 86.35%, respectively. Group 1 had low probabilities of systolic and diastolic hypertension and remained stable during the whole follow-up. Group 2 started with low probabilities, with both SBP and DBP gradually increasing thereafter. SBP and DBP decreased sharply in trajectory 3. Group 4 was characterized by approximately inverted U shapes; it had moderate probabilities of systolic and diastolic hypertension at baseline, then increased, and finally decreased to moderate levels. Group 5 had a high probability of systolic hypertension but a low probability of diastolic hypertension; these trajectory curves had downward trends. Group 6 had persistently high probabilities of systolic and diastolic hypertension.

Table 1 presents the baseline patient characteristics grouped by multi-trajectories of systolic and diastolic hypertension. The proportions of CHD differed significantly between the six trajectory groups (2.68, 3.99, 5.76, 7.01, 8.04, and 7.83%, respectively; *P* < 0.001). The CHD incidence densities were 3.89, 5.58, 8.48, 9.61, 11.35, and 11.07 per 1,000 person-years, respectively. Significant differences in baseline age, gender, smoking, drinking, BMI, LDL-C, HDL-C, TG, FPG, SBP, DBP, high SBP rate, and high DBP rate were observed (*P* < 0.001). Overall, the group with the ISH phase had a higher prevalence of CHD, age, and FPG, people in the low-stable group were more likely to be women, and the proportions of smoking and drinking were higher in both groups 4 and 6. BMI, SBP, DBP, LDL-C, TG, high SBP rate, and high DBP rate were all higher in group 6. Groups 1 and 4 had higher HDL-C.

**Table 1:** Baseline characteristics grouped by different multi-trajectories of systolic and diastolic hypertension.

**Characteristics**	**Group 1**	**Group 2**	**Group 3**	**Group 4**	**Group 5**	**Group 6**	***P***
Sample size, *n*	2,050	651	434	385	199	383	–
Incident CHD, *n* (%)	55 (2.68)	26 (3.99)	25 (5.76)	27 (7.01)	16 (8.04)	30 (7.83)	< 0.001
CHD incidence density, per 1,000 person-years	3.89	5.58	8.48	9.61	11.35	11.07	–
Age, year	43.00 (8.00)	45.00 (11.00)	46.00 (10.00)	45.00 (10.00)	60.00 (15.00)	48.00 (12.50)	< 0.001
Female, *n* (%)	1,032 (50.30)	168 (25.80)	95 (21.90)	46 (11.90)	50 (25.10)	33 (8.60)	< 0.001
Smoking, *n* (%)	358 (17.50)	134 (20.60)	106 (24.40)	101 (26.20)	33 (16.60)	109 (28.50)	< 0.001
Drinking, *n* (%)	693 (33.80)	319 (49.00)	240 (55.30)	255 (66.20)	80 (40.20)	256 (66.80)	< 0.001
BMI, kg/m^2^	23.70 ± 2.90	25.10 ± 3.00	26.10 ± 2.90	25.80 ± 3.00	25.60 ± 3.10	26.90 ± 3.00	< 0.001
LDL-C, mmol/L	2.90 ± 0.70	3.00 ± 0.70	3.10 ± 0.70	3.00 ± 0.70	3.00 ± 0.80	3.10 ± 0.70	< 0.001
HDL-C, mmol/L	1.40 ± 0.30	1.30 ± 0.30	1.30 ± 0.30	1.40 ± 0.30	1.30 ± 0.30	1.30 ± 0.30	< 0.001
TG, mmol/L	1.10 (0.80)	1.30 (1.10)	1.60 (1.30)	1.60 (1.40)	1.40 (1.20)	1.80 (1.50)	< 0.001
FPG, mmol/L	5.10 (0.80)	5.30 (0.80)	5.40 (0.90)	5.30 (0.80)	5.70 (1.20)	5.50 (1.10)	< 0.001
SBP, mmHg	117.50 ± 11.70	126.50 ± 11.00	144.00 ± 12.80	136.00 ± 13.00	151.20 ± 14.30	158.00 ± 15.80	< 0.001
DBP, mmHg	73.40 ± 8.80	79.80 ± 8.20	94.00 ± 7.90	88.90 ± 9.40	83.90 ± 8.70	102.50 ± 9.60	< 0.001
High SBP, *n* (%)	43 (2.10)	51 (7.80)	294 (67.70)	129 (33.50)	170 (85.40)	364 (95.00)	< 0.001
High DBP, *n* (%)	47 (2.30)	41 (6.30)	358 (82.50)	187 (48.60)	47 (23.60)	363 (94.80)	< 0.001

Data are presented as means ± SDs, median (IQR), and numbers or numbers (%). Six trajectory groups are presented in Figure 1. CHD, coronary heart disease; BMI, body mass index; LDL-C, low-density lipoprotein cholesterol; HDL-C, high-density lipoprotein cholesterol; TG, triglyceride; FPG, fasting plasma glucose; SBP, systolic blood pressure; DBP, diastolic blood pressure.

**Figure 2:**
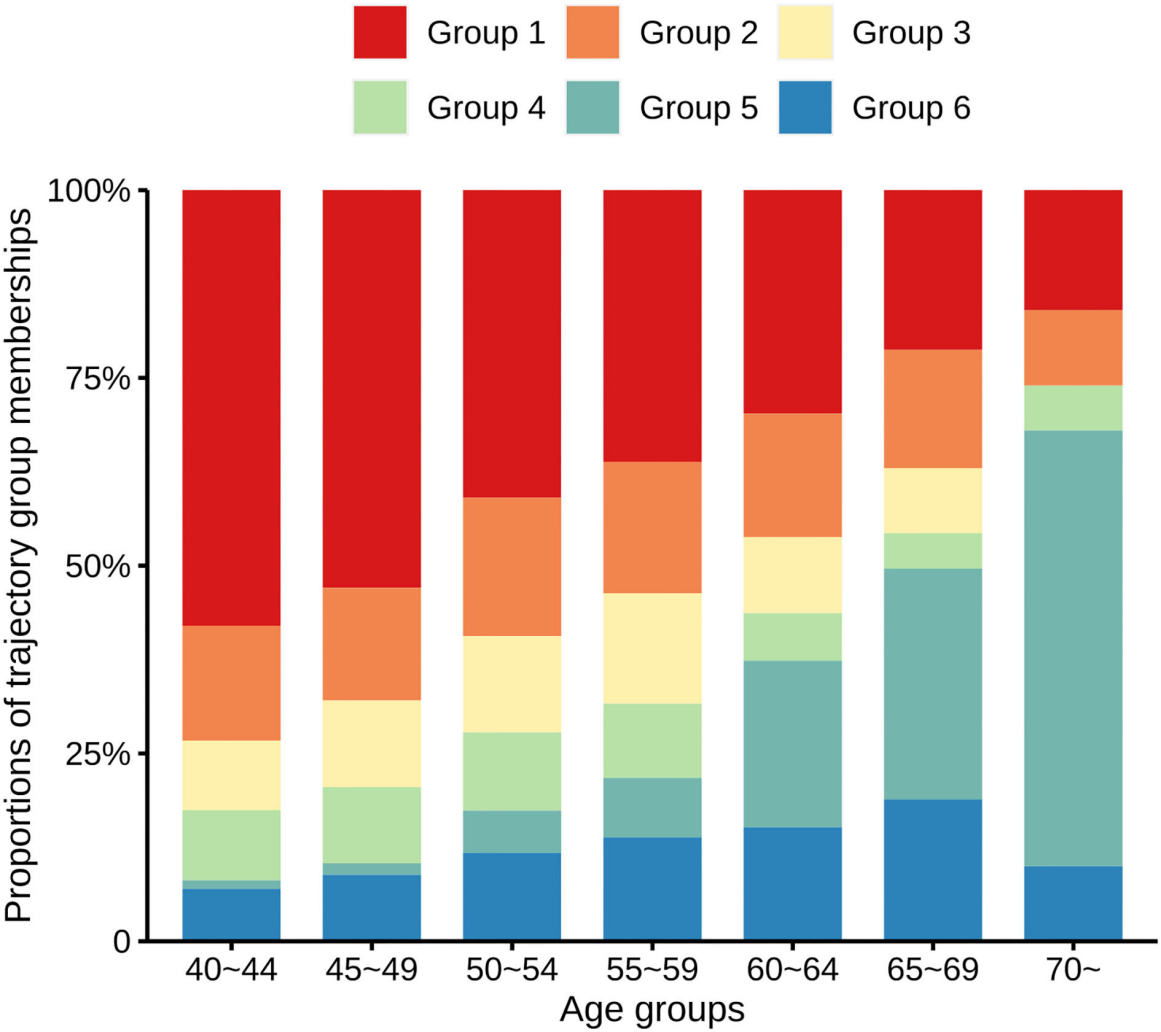
Proportions of the trajectory group memberships in distinct age groups. Bar charts of different colors represent the proportions of six different multi-trajectories of systolic and diastolic hypertension.

Figure 2 shows the proportions of each age group for each trajectory. The proportions of participants in the low-stable group gradually decreased as their ages increased, being 57.99% in the 40–44 age group, 52.95% in the 45–49 age group, 40.96% in the 50–54 age group, 36.16% in the 55–59 age group, 29.75% in the 60–64 age group, 21.26% in the 65–69 age group, and 16.00% in the ≥70 age group. In contrast, the proportions of participants in the ISH group increased with increasing age, being 1.12% in the 40–44 age group, 1.54% in the 45–49 age group, 5.63% in the 50–54 age group, 7.91% in the 55–59 age group, 22.15% in the 60–64 age group, 30.71% in the 65–69 age group, and 58.00% in the ≥70 age group, respectively.

Multivariate logistic regression analysis was conducted with various reference groups to determine the effects of the trajectory group on the risk of CHD. We found that using group 1 or 2 as the reference group covered all significant findings (Supplementary Table S3). We, therefore, specified these as reference groups for further analysis. Odds ratios (ORs) and 95% confidence interval (CIs) for the effects of multi-trajectories on the risk of CHD compared with trajectory group 1 are shown in Table 2. After adjustment for baseline covariates including age, gender, smoking, drinking, BMI, LDL-C, HDL-C, TG, and FPG, the ORs (95% CIs) for groups 4 and 6 were 2.23 (1.34–3.70) and 1.87 (1.12–3.11), respectively. The ORs and 95% CIs for the effects of multi-trajectories on the risk of CHD compared with trajectory group 2 are shown in Table 3. The ORs (95% CIs) for group 4 were 1.88 (1.06–3.31) after adjustment for baseline covariates.

**Table 2:** ORs and 95% CIs of multi-trajectories on CHD risk compared with trajectory group 1.

**Covariates**	**Model 1**	**Model 2**
Group 1	Reference	Reference
Group 2	1.51 (0.94–2.43)	1.19 (0.73–1.94)
Group 3	2.22 (1.37–3.60)^†^	1.63 (0.98–2.71)
Group 4	2.74 (1.70–4.40)^*^	2.23 (1.34–3.70)^†^
Group 5	3.17 (1.78–5.65)^*^	1.14 (0.60–2.19)
Group 6	3.08 (1.95–4.88)^*^	1.87 (1.12–3.11)^‡^
Age		1.07 (1.05–1.09)^*^
**Gender**		
Male		Reference
Female		1.21 (0.77–1.92)
Smoking		1.67 (1.16–2.42)^†^
Drinking		0.92 (0.63–1.36)
BMI		1.07 (1.01–1.13)^‡^
LDL-C		1.32 (1.08–1.62)^†^
HDL-C		1.40 (0.85–2.28)
TG		1.04 (0.90–1.20)
FPG		1.00 (0.89–1.12)

ORs, odds ratios; CIs, confidence intervals; CHD, coronary heart disease; BMI, body mass index; LDL-C, low-density lipoprotein cholesterol; HDL-C, high-density lipoprotein cholesterol; TG, triglyceride; FPG, fasting plasma glucose.

Model 1 is adjusted for trajectory group.

Model 2 is adjusted for variables in model 1 plus age, gender, smoking, drinking, BMI, LDL-C, HDL-C, TG, and FPG.

* P < 0.001;

† P < 0.01;

‡ P < 0.05.

**Table 3:** ORs and 95% CIs of multi-trajectories on CHD risk compared with trajectory group 2.

**Covariates**	**Model 1**	**Model 2**
Group 2	Reference	Reference
Group 1	0.66 (0.41–1.07)	0.84 (0.52–1.38)
Group 3	1.47 (0.84–2.58)	1.37 (0.77–2.43)
Group 4	1.81 (1.04–3.15)^‡^	1.88 (1.06–3.31)^‡^
Group 5	2.10 (1.10–4.00)^‡^	0.96 (0.48–1.92)
Group 6	2.04 (1.19–3.51)^†^	1.58 (0.90–2.76)
Age		1.07 (1.05–1.09)^*^
**Gender**		
Male		Reference
Female		1.21 (0.77–1.92)
Smoking		1.67 (1.16–2.42)^†^
Drinking		0.92 (0.63–1.36)
BMI		1.07 (1.01–1.13)^‡^
LDL-C		1.32 (1.08–1.62)^†^
HDL-C		1.40 (0.85–2.28)
TG		1.04 (0.90–1.20)
FPG		1.00 (0.89–1.12)

ORs, odds ratios; CIs, confidence intervals; CHD, coronary heart disease; BMI, body mass index; LDL-C, low-density lipoprotein cholesterol; HDL-C, high-density lipoprotein cholesterol; TG, triglyceride; FPG, fasting plasma glucose.

Model 1 is adjusted for trajectory group.

Model 2 is adjusted for variables in model 1 plus age, gender, smoking, drinking, BMI, LDL-C, HDL-C, TG, and FPG.

* P < 0.001;

† P < 0.01;

‡ P < 0.05.

Multinomial logistic analysis was conducted to assess the impact of baseline covariates on multi-trajectories (Tables 4, 5). Age, gender, BMI, and FPG were independent risk factors for group 2. Age, gender, BMI, TG, and FPG were independent predictors for group 3. Age, gender, drinking, BMI, TG, and FPG were independently associated with group 4 and group 6. Age, BMI, TG, and FPG showed an independent impact on group 5. In the above significant covariates, gender showed a negative correlation with multi-trajectories while other variables showed a positive association with trajectory groups.

**Table 4:** ORs and 95% CIs of single covariates on multi-trajectories with trajectory group 1 as reference.

**Covariates**	**Group 2**	**Group 3**	**Group 4**	**Group 5**	**Group 6**
Age	1.04 (1.03–1.06)^*^	1.05 (1.03–1.06)^*^	1.03 (1.02–1.05)^*^	1.20 (1.18–1.23)^*^	1.08 (1.07–1.10)^*^
**Gender**					
Male	Reference	Referenc	Reference	Reference	Reference
Female	0.34 (0.28–0.42)^*^	0.28 (0.22–0.35)^*^	0.13 (0.10–0.18)^*^	0.33 (0.24–0.46)^*^	0.09 (0.06–0.13)^*^
Smoking	1.23 (0.98–1.53)	1.53 (1.19–1.96)^*^	1.68 (1.30–2.17)^*^	0.94 (0.64–1.39)	1.88 (1.47–2.41)^*^
Drinking	1.88 (1.57–2.25)^*^	2.42 (1.96–2.99)^*^	3.84 (3.05–4.84)^*^	1.32 (0.98–1.77)	3.95 (3.13–4.98)^*^
BMI	1.18 (1.15–1.22)^*^	1.32 (1.27–1.37)^*^	1.28 (1.23–1.33)^*^	1.25 (1.19–1.32)^*^	1.42 (1.37–1.48)^*^
LDL-C	1.29 (1.14–1.45)^*^	1.49 (1.29–1.71)^*^	1.36 (1.17–1.58)^*^	1.37 (1.13–1.67)^†^	1.55 (1.34–1.79)^*^
HDL-C	0.67 (0.51–0.89)^†^	0.46 (0.33–0.64)^*^	0.88 (0.63–1.22)	0.64 (0.41–1.010)	0.47 (0.33–0.67)^*^
TG	1.33 (1.22–1.46)^*^	1.68 (1.54–1.84)^*^	1.71 (1.55–1.87)^*^	1.43 (1.25–1.63)^*^	1.79 (1.63–1.96)^*^
FPG	1.35 (1.23–1.49)^*^	1.54 (1.40–1.69)^*^	1.46 (1.31–1.61)^*^	1.78 (1.61–1.96)^*^	1.63 (1.49–1.79)^*^

ORs, odds ratios; CIs, confidence intervals; CHD, coronary heart disease; BMI, body mass index; LDL-C, low-density lipoprotein cholesterol; HDL-C, high-density lipoprotein cholesterol; TG, triglyceride; FPG, fasting plasma glucose.

* P < 0.001;

† P < 0.01.

**Table 5:** ORs and 95% CIs of multiple covariates on multi-trajectories with trajectory group 1 as reference.

**Models**	**Covariates**	**Group 2**	**Group 3**	**Group 4**	**Group 5**	**Group 6**
Model 1	Age	1.04 (1.02–1.05)^*^	1.04 (1.02–1.05)^*^	1.03 (1.01–1.05)^*^	1.20 (1.17–1.22)^*^	1.08 (1.07–1.10)^*^
	Smoking	0.83 (0.66–1.06)	0.90 (0.69–1.18)	0.88 (0.67–1.16)	0.69 (0.45–1.07)	0.94 (0.71–1.24)
	Drinking	1.67 (1.37–2.03)^*^	1.84 (1.46–2.32)^*^	2.90 (2.26–3.72)^*^	1.65 (1.16–2.33)^†^	3.15 (2.43–4.09)^*^
	BMI	1.13 (1.10–1.17)^*^	1.23 (1.18–1.28)^*^	1.20 (1.15–1.25)^*^	1.19 (1.13–1.26)^*^	1.33 (1.28–1.39)^*^
	LDL–C	1.08 (0.94–1.22)	1.16 (0.99–1.35)	1.04 (0.88–1.21)	0.90 (0.72–1.12)	1.12 (0.95–1.32)
	HDL–C	0.98 (0.73–1.33)	0.91 (0.64–1.32)	1.71 (1.19–2.46)^†^	1.62 (0.97 2.69)	1.15 (0.78–1.70)
	TG	1.08 (0.98–1.19)	1.28 (1.16–1.41)^*^	1.32 (1.20–1.46)^*^	1.25 (1.08–1.46)^†^	1.33 (1.20–1.47)^*^
	FPG	1.11 (1.01–1.21)^‡^	1.18 (1.07–1.29)^*^	1.13 (1.02–1.26)^‡^	1.30 (1.17–1.44)^*^	1.19 (1.09–1.31)^*^
Model 2	Age	1.03 (1.01–1.04)^*^	1.03 (1.01–1.05)^*^	1.02 (1.01–1.04)^‡^	1.19 (1.17–1.22)^*^	1.07 (1.06–1.09)^*^
	**Gender**					
	Male	Reference	Reference	Reference	Reference	Reference
	Female	0.45 (0.34–0.58)^*^	0.53 (0.38–0.72)^*^	0.25 (0.17–0.36)^*^	0.69 (0.45–1.08)	0.23 (0.15–0.35)^*^
	Drinking	0.99 (0.79–1.25)	1.21 (0.92–1.60)	1.38 (1.04–1.84)^‡^	1.16 (0.78–1.71)	1.57 (1.17–2.11)^†^
	BMI	1.12 (1.08–1.16)^*^	1.22 (1.17–1.27)^*^	1.18 (1.13–1.23)^*^	1.19 (1.12–1.26)^*^	1.31 (1.26–1.37)^*^
	LDL-C	1.07 (0.95–1.22)	1.16 (0.99–1.34)	1.03 (0.87–1.20)	0.89 (0.71–1.11)	1.11 (0.95–1.31)
	HDL-C	1.14 (0.84–1.54)	1.02 (0.71–1.48)	2.06 (1.43–2.97)^*^	1.69 (1.01–2.85)^‡^	1.42 (0.96–2.10)
	TG	1.04 (0.95–1.15)	1.25 (1.13–1.37)^*^	1.28 (1.16–1.41)^*^	1.22 (1.05–1.42)^†^	1.29 (1.16–1.42)^*^
	FPG	1.10 (1.01–1.20)^‡^	1.17 (1.06–1.28)^†^	1.12 (1.01–1.24)^‡^	1.29 (1.17–1.43)^*^	1.18 (1.07–1.29)^*^

ORs, odds ratios; CIs, confidence intervals; CHD, coronary heart disease; BMI, body mass index; LDL-C, low-density lipoprotein cholesterol; HDL-C, high-density lipoprotein cholesterol; TG, triglyceride; FPG, fasting plasma glucose.

Model 1 is the multinomial logistic model adjusting age, smoking, drinking, BMI, LDL-C, HDL-C, TG, and FPG.

Model 2 is the multinomial logistic model adjusting age, gender, drinking, BMI, LDL-C, HDL-C, TG, and FPG.

* P < 0.001;

† P < 0.01;

‡ P < 0.05.

According to the univariate analysis, the optimal number of trajectories for both systolic and diastolic hypertension separately was three (Supplementary Figure S1). Univariate GBTM did not identify elevated or ISH trend curves. The model screening process is shown in Supplementary Table S4. After adjustment for baseline age, gender, smoking, drinking, BMI, LDL-C, HDL-C, TG, and FPG, the univariate analysis indicated that trajectories of diastolic hypertension were not independently associated with the risk of CHD (Supplementary Table S5). Trajectory group 3 for systolic hypertension had a relatively higher risk of CHD after adjustment for multiple variables.

## Discussion

In this retrospective cohort study of healthy middle-aged and older adults, we identified six multi-trajectories of systolic and diastolic hypertension and identified some independent associations between the trajectory group membership and the risk of CHD. We found that the medium-increasing-decreasing group (group 4) and the high-decreasing group (group 6) were critical periods for the development of CHD. Age, gender, drinking, BMI, TG, and FPG were independent predictors for trajectory groups 4 and 6. Using a multi-trajectory model enabled the identification of synergistic changes in SBP and DBP that are potentially correlated with the risk of CHD. To the best of our knowledge, this is the first study to investigate the multi-trajectories of systolic and diastolic hypertension and their association with the risk of CHD.

Effective early and long-term management of hypertension is aimed at preventing transition to adverse types of multi-trajectories and is especially indicated for individuals with certain trajectories. Of the 4,102 participants, 25.62% had a high SBP and 25.43% had a high DBP at baseline. However, about 50.02% of participants (groups 2–6) developed hypertension during the follow-up period. Interestingly, the six trajectory groups that we identified reflected different stages of the development of hypertension. The process of developing hypertension from having a normal BP is a chronic one, comprising moving from constant normal to abnormal, and then to persistent abnormal. Different stages of hypertension need to be controlled to reduce the risk of CHD. Individuals can check their BP daily, weekly, monthly, or yearly. We allocated individuals to trajectory groups on the basis of easily obtained, longitudinal BP measurements. Individuals with low-stable trajectories need to avoid transitioning to the low-increasing group, whereas those with low-increasing trajectories are at potential risk of developing into the medium-increasing-decreasing group, which ideally be detected early. Those with medium-decreasing trajectories need to maintain that status, whereas those with medium-increasing-decreasing trajectories are at high risk of developing CHD. Individuals at the latter stage of hypertension need to be identified early and preventive measures should be instituted to promote transitioning to a medium-decreasing trajectory. In individuals with ISH, attention should be paid to the high levels of SBP and the rebound that eventually leads to ISH being avoided. Individuals suffering from sustained abnormal SBP and DBP have already missed the optimal stage for preventing hypertension and reducing the risk of CHD, but still require the implementation of long-term, effective management of BP.

It is necessary to explore multi-trajectories of BP because they represent distinct stages of hypertension. We found low-stable and low-increasing trajectories to both be negatively associated with CHD compared with a group with medium-increasing-decreasing trajectories. Individuals with the low-stable group do not have hypertension, whereas those with the low-increasing group are in the early stage of hypertension. It should be noticed that, during the initial phase of follow-up, the medium-decreasing group (group 3) had higher BPs than did the medium-increasing-decreasing group (group 4), but thereafter the BP level of group 4 increased whereas those of group 3 decreased. Moreover, group 4, who are in an adverse progression stage of hypertension, was younger, had lower BMI, LDL-C and FPG, and had higher HDL-C than group 3, which indicated people in group 4 were healthier at baseline. Individuals in Group 3 are recovering from hypertension. We identified a medium-increasing-decreasing trajectory (Group 4) as denoting a critical stage of transition from health to adverse outcomes. It is, therefore, important to determine whether an individual has a medium-increasing-decreasing trajectory early. Interestingly, individuals in group 5 tended to develop ISH. To our knowledge, we were unable to find similar ISH tendencies in any previous reports about BP trajectories. Multi-trajectories reflect a combination of variations in systolic and diastolic hypertension. In the present study, older individuals were more likely to have an ISH trajectory (Figure 2) which is consistent with the reality that a high proportion of older patients with hypertension have ISH (27). Several studies showed that, in middle-aged and older individuals, reduction in DBP is an adverse finding, being associated with an increased risk of CHD (6, 28). Moreover, the combination of a very high SBP and moderate DBP levels also contributes to cardiovascular disease (29). Therefore, the levels of SBP and DBP should be managed early and balanced to prevent them from developing into persistent ISH states. We found that a high-decreasing trajectory is independently associated with the risk of CHD, confirming that long exposure to uncontrolled BP has a cumulative effect and contributes to the development of CHD.

Age, gender, drinking, BMI, TG, and FPG were driver predictors for groups 4 and 6, and the findings indicated that these variables need to be emphasized in the prevention stages to early change in the development trend of hypertension status. In addition, age, BMI, and FPG were found to be common factors for the abnormal changing process of hypertension (groups 2, 3, 4, 5, and 6), which were worthy of attention and exploration in further studies.

More recent studies have focused on univariate BP trajectory patterns (7–12). The multi-trajectory model used in the present study enabled the identification of diverse stages of hypertension with clinical significance. Norby et al. reported five trajectories of SBP, which mainly showed a longitudinal change (9). Portegies et al. fitted four trajectories of SBP ranging from 55 to 106 years (10). Zheng et al. reported four trajectories each for SBP and DBP but did not identify downward curves (11). Norby et al. and Sharashova et al. separately identified four and three hypertension trajectories, respectively, without clear downward trends (8, 9). A downward trajectory indicates that interventions to combat hypertension have been effective. We also fitted univariate trajectories of systolic hypertension and diastolic hypertension. It is not surprising that univariate trajectories were inferior to multivariate trajectories in curve diversity and independent associations with the risk of CHD. Multivariate GBTM allows for potential synergistic effects of longitudinal change of systolic and diastolic hypertension and fits novel, reasonable, and clinically defined trajectories. Stable, slowly increasing, rapidly increasing, and distinct decreasing trajectories reflected diverse stages of hypertension. The multivariate hypertension trajectories identified in this study are explainable and seemed to conform with clinical realities. The multivariate trajectory model also has the advantage of enabling exploration of the co-evolution of blood lipids (17) and adverse childhood events (30).

The strengths of this study included the availability of repeated measurements, multi-trajectories of systolic and diastolic hypertension, and robust results after adjustment for various covariates. However, the following limitations need to be highlighted. First, the findings were based on self-reported data and so cannot be directly extended to other cohorts. Second, physical activities and antihypertensive drugs were not considered for inclusion in the study due to insufficient information. Third, methods in this observational study were unable to make causal inferences between multi-trajectories and CHD. In addition, the ascertainment of CHD cases may not be complete.

In conclusion, we have identified six innovative, clinically defined multi-trajectories of systolic and diastolic hypertension that represent distinct stages of hypertension. We then demonstrated the independent association between such trajectories and CHD risk. For middle-aged and older adults, the medium-increasing decreasing and high-decreasing groups are potentially critical periods for the development of CHD. Preventing adverse changes in hypertension status and reducing the high risk of CHD is necessary for people in distinct trajectory groups. Future studies are needed to investigate how to use available indicators to predict long-term trajectories early, enabling the translation of findings about trajectories into medical practice.

## Data availability statement

The datasets presented in this article are not readily available because of privacy policy. Requests to access the datasets should be directed to yuanzhongshang@sdu.edu.cn.

## Ethics statement

The studies involving human participants were reviewed and approved by the Ethics Committee of the School of Public Health, Shandong University (No. 20031112). The patients/participants provided their written informed consent to participate in this study.

## Author contributions

ML and ZY conceived the study and drafted the manuscript. MZ, YY, YL, and CW helped to manage the data. ML, YZ, ZY, and XS performed the statistical analysis and to helped interpret the results. CY, WG, FT, and XS checked the spelling and grammar of the paper. FX has access to the data in this study. All authors contributed to the article and approved the submitted version.

## Funding

This study was funded by the National Natural Science Foundation of China (81872712, 81673272), the Natural Science Foundation of Shandong Province (ZR2019ZD02), the Cheeloo Young Talent Program of Shandong University, and the Taishan Scholar Project of Shandong Province. The funding body played no role in the design of the study, collection, analyses, and interpretation of data and in writing the article.

## Conflict of interest

The authors declare that the research was conducted in the absence of any commercial or financial relationships that could be construed as a potential conflict of interest.

## Publisher's note

All claims expressed in this article are solely those of the authors and do not necessarily represent those of their affiliated organizations, or those of the publisher, the editors and the reviewers. Any product that may be evaluated in this article, or claim that may be made by its manufacturer, is not guaranteed or endorsed by the publisher.

## Abbreviations

BP: blood pressure
CHD: coronary heart disease
SBP: systolic blood pressure
DBP: diastolic blood pressure
GBTM: group-based trajectory model
BMI: body mass index
LDL-C: low-density lipoprotein cholesterol
HDL-C: high-density lipoprotein cholesterol
TG: triglyceride
FPG: fasting blood glucose
ICD-10: 10th revision International Classification of Diseases
SDs: standard deviations
BIC: Bayesian information criterion
ISH: isolated systolic hypertension
OR: odds ratio
CIs: confidence intervals.
