# Supplementary material for: Multi-trajectories of systolic and diastolic hypertension and coronary heart disease in middle-aged and older adults
Source: Front Public Health. 2022 Nov 24;10:1017727. doi: 10.3389/fpubh.2022.1017727 (PMC9729777; doi:10.3389/fpubh.2022.1017727)
Supplement: Supplementary file 1 [file Data_Sheet_1.docx]

**Supplementary**

Table S1. The model fitting of multi-trajectories of systolic and diastolic hypertension.

Table S2. Parameter estimates for the optimal group-based multi-trajectory model.

Table S3. ORs and 95% CIs of trajectory group on CHD risk with different reference groups.

Table S4. The model fitting of the univariate trajectories of systolic and diastolic hypertension.

Figure S1. Univariate trajectories of systolic and diastolic hypertension.

Table S5. ORs and 95% CIs of univariate trajectories of systolic and diastolic hypertension on CHD risk.

Table S1. The model fitting of multi-trajectories of systolic and diastolic hypertension

| Number of groups | Variables | Highest order of trajectory curve | BIC | % Participants | Mean posterior probabilities |
| --- | --- | --- | --- | --- | --- |
| 1 | Systolic hypertension  Diastolic hypertension | 2  2 | -25527.53 | 100% | 100% |
| 2 | Systolic hypertension  Diastolic hypertension | 2/2  2/2 | -19300.93 | 67.31%/32.69% | 97.99%/96.59% |
| 3 | Systolic hypertension  Diastolic hypertension | 2/2/2  2/2/2 | -18647.10 | 55.95%/28.91%/15.14% | 94.85%/88.58%/92.40% |
| 4 | Systolic hypertension  Diastolic hypertension | 2/2/2/2  2/2/2/2 | -18527.89 | 45.59%/25.67%/  19.72%/9.02% | 90.54%/83.45%/  83.68%/86.16% |
| 5 | Systolic hypertension  Diastolic hypertension | 2/2/2/2/2  2/2/2/2/2 | -18376.86 | 55.68%/11.24%/4.34%/  12.82%/15.92% | 93.69%/75.90%/80.83%/  77.43%/90.09% |
| **6** | **Systolic hypertension**  **Diastolic hypertension** | **2/2/2/2/2/2**  **2/2/2/2/2/2** | **-18303.36** | **49.98%/15.87%/10.58%/**  **9.39%/4.85%/9.34%** | **89.47%/72.12%/75.51%/**  **75.61%/78.52%/86.35%** |
| 7 | Systolic hypertension  Diastolic hypertension | 2/2/2/2/2/2/2  2/2/2/2/2/2/2 | Unable to calculate parameters | | |

BIC indicates Bayesian information criterion; the optimal model is highlighted in bold.

Table S2. Parameter estimates for the optimal group-based multi-trajectory model

| Variables | Group | Coefficient of trajectory equations | | |
| --- | --- | --- | --- | --- |
|  |  | Intercept | Linear | Quadratic |
| Systolic hypertension | 1 | -4.040 | -0.079 | 0.005 |
|  | 2 | -2.409 | 0.276 | -0.010 |
|  | 3 | 1.089 | -0.666 | 0.025 |
|  | 4 | -1.077 | 0.621 | -0.059 |
|  | 5 | 1.308 | 0.240 | -0.038 |
|  | 6 | 2.931 | -0.146 | -0.007 |
| Diastolic hypertension | 1 | -3.704 | -0.388 | 0.036 |
|  | 2 | -2.191 | 0.202 | -0.010 |
|  | 3 | 1.763 | -0.808 | 0.034 |
|  | 4 | -0.500 | 0.648 | -0.069 |
|  | 5 | -0.850 | -0.126 | -0.002 |
|  | 6 | 2.902 | -0.301 | 0.007 |

Group 1 to group 6 indicate different multi-trajectories of systolic and diastolic hypertension.

Table S3. ORs and 95% CIs of trajectory group on CHD risk with different reference groups

| Reference | Subgroup | Model |
| --- | --- | --- |
| Group 1 | Group 2 | 1.19 (0.73-1.94) |
|  | Group 3 | 1.63 (0.98-2.71) |
|  | Group 4 | 2.23 (1.34-3.70)† |
|  | Group 5 | 1.14 (0.60-2.19) |
|  | Group 6 | 1.87 (1.12-3.11)‡ |
| Group 2 | Group 1 | 0.84 (0.52-1.38) |
|  | Group 3 | 1.37 (0.77-2.43) |
|  | Group 4 | 1.88 (1.06-3.31)‡ |
|  | Group 5 | 0.96 (0.48-1.92) |
|  | Group 6 | 1.58 (0.90-2.76) |
| Group 3 | Group 1 | 0.61 (0.37-1.02) |
|  | Group 2 | 0.73 (0.41-1.30) |
|  | Group 4 | 1.37 (0.77-2.43) |
|  | Group 5 | 0.70 (0.35-1.42) |
|  | Group 6 | 1.15 (0.65-2.02) |
| Group 4 | Group 1 | 0.45 (0.27-0.75)† |
|  | Group 2 | 0.53 (0.30-0.94)‡ |
|  | Group 3 | 0.73 (0.41-1.30) |
|  | Group 5 | 0.51 (0.25-1.04) |
|  | Group 6 | 0.84 (0.48-1.46) |
| Group 5 | Group 1 | 0.87 (0.46-1.67) |
|  | Group 2 | 1.04 (0.52-2.06) |
|  | Group 3 | 1.42 (0.71-2.86) |
|  | Group 4 | 1.95 (0.96-3.93) |
|  | Group 6 | 1.63 (0.83-3.20) |

Group 1 to group 6 indicate different multi-trajectories of systolic and diastolic hypertension.

Model is adjusted for age, gender, smoking, drinking, BMI, LDL-C, HDL-C, TG and FPG.

**P*<0.001; †*P*<0.01; ‡*P*<0.05.

Table S4. The model fitting of the univariate trajectories of systolic and diastolic hypertension

| Variables | Number of groups | Highest order of trajectory curve | BIC | % Participants | Mean posterior probabilities |
| --- | --- | --- | --- | --- | --- |
| Systolic hypertension | 1 | 2 | -13031.22 | 100% | 100% |
|  | 2 | 2/2 | -10171.47 | 71.11%/28.89% | 97.65%/93.14% |
|  | **3** | **2/2/2** | **-10020.39** | **58.36%/25.48%/16.16%** | **91.48%/78.45%/86.58%** |
|  | 4 | 2/2/2/2 | -10017.77 | 63.04%/13.58%/  7.34%/16.04% | 88.67%/63.98%/  63.21%/84.76% |
| Diastolic hypertension | 1 | 2 | -12496.31 | 100% | 100% |
|  | 2 | 2/2 | -9924.00 | 70.87%/29.13% | 96.82%/94.14% |
|  | **3** | **2/2/2** | **-9802.25** | **62.97%/24.28%/12.75%** | **92.31%/77.64%/83.17%** |
|  | 4 | 2/2/2/2 | -9796.54 | 64.36%/15.41%/  5.92%/14.31% | 89.79%/63.10%/  59.90%/81.79% |

BIC indicates Bayesian information criterion; the optimal model is highlighted in bold.


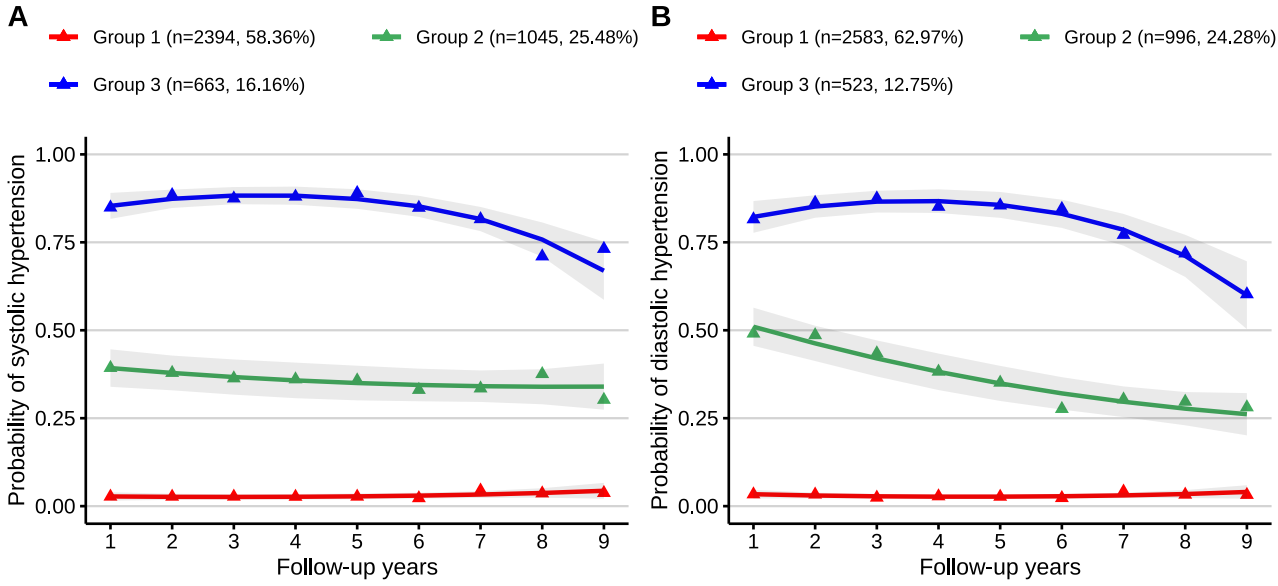


Figure S1. Univariate trajectories of systolic and diastolic hypertension

Figure S1 shows univariate trajectories of systolic and diastolic hypertension which were identified from the GBTM. Gray shadows represent the 95% confidence intervals for each trajectory; the solid lines represent expected trajectories; the points represent observed trajectories; numbers and proportions of group memberships are listed.

Table S5. ORs and 95% CIs of univariate trajectories of systolic and diastolic hypertension on CHD risk

| Systolic hypertension | | |  | Diastolic hypertension | | |
| --- | --- | --- | --- | --- | --- | --- |
| Model | Subgroup | ORs and 95% CIs |  | Model | Subgroup | ORs and 95% CIs |
| Model 1 | Group 1 | Reference |  | Model 1 | Group 1 | Reference |
|  | Group 2 | 1.93 (1.34-2.78)* |  |  | Group 2 | 0.68 (0.47-0.96)‡ |
|  | Group 3 | 3.27 (2.27-4.70)* |  |  | Group 3 | 1.57 (1.02-2.41)‡ |
| Model 2 | Group 1 | Reference |  | Model 2 | Group 1 | Reference |
|  | Group 2 | 1.40 (0.95-2.05) |  |  | Group 2 | 0.79 (0.54-1.14) |
|  | Group 3 | 1.76 (1.16-2.67)† |  |  | Group 3 | 1.50 (0.97-2.34) |

ORs indicates odds ratios; CIs, confidence intervals; CHD, coronary heart disease. Group 1 to group 3 indicate different univariate trajectories of systolic and diastolic hypertension.

Model 1 is adjusted for trajectory group;

Model 2 is adjusted for variables in model 1 plus age, gender, smoking, drinking, BMI, LDL-C, HDL-C, TG and FPG.

**P*<0.001; †*P*<0.01; ‡*P*<0.05.
